# Comparison of HPLC and UV Spectrophotometric Methods for Quantification of Favipiravir in Pharmaceutical Formulations

**DOI:** 10.22037/ijpr.2020.114199.14725

**Published:** 2021

**Authors:** İbrahim Bulduk

**Keywords:** Favipiravir, Analysis, Method, Development, Validation, Comparative

## Abstract

There is no currently successful method to treat Covid-19 infection. Nevertheless, previously licensed pharmaceuticals to treat other virus infections are used on an off-label basis either alone or in combination. One of them is favipiravir. Favipiravir, also known as favilavir, is an antiviral drug that is active against many viruses. Spectrophotometric and liquid chromatographic methods have been developed and validated for the quantitative determination of favipiravir in pharmaceutical formulations. Chromatographic method has been performed using reverse-phase technique on a C-18 column with a mobile phase consisting of sodium acetate solution (pH adjusted to 3.0 with glacial acetic acid) and acetonitrile (85:15, v/v) at 30 ^o^C. The mobile phase flow rate was 1.0 mL min^-1^. For the determination of favipiravir, UV spectrum has been recorded between 200 and 800 nm using deionized water as solvent and the wavelength of 227 nm has been selected. Both methods have been validated in terms of their specificity, linearity, limits of detection and quantification, precision, accuracy, and robustness. Both methods have demonstrated good linearity, precision and recovery. No spectral and chromatographic interferences from the tablet excipients were found in spectrophotometric and liquid chromatographic methods. In both methods, correlation coefficients were greater than 0.999 within a concentration range of 10–60 mg mL-^1^ using spectrophotometry and chromatography. Intra-day and inter-day precision were observed with low relative standard deviation values. The accuracy of the methods were within the range 99.57-100.10% for LC and from 99.83–100.45% for UV. Therefore, both methods gave the most reliable outcomes for the determination of favipiravir in pharmaceutical formulation.

## Introduction

The number of cases of Covid-19 would certainly be in the millions, according to the latest WHO report. According to data revised on 28 September 2020: more than 995 thousand deaths and more than 32 million worldwide cases (1).

The intermediate host of Covid-19 is unknown yet to date, and there is no successful treatment method to treat Covid-19 infection. Currently, some pharmaceutical agents are used on an off-label basis, either alone or in combination. However, we need more evidence and confirmation to achive a gold standard clinical treatment with high effectiveness and low side effect. Several Covid-19 clinical trials are currently underway on previously licensed pharmaceuticals to treat other virus infections (2).

Bromhexine, Arbidol, Hydroxychloroquine, Ruxolitinib, Baricitinib, Oseltamivir, Emtricitabine, Atazanavir, Lopinavir, Darunavir, Danoprevir, Noscapine, Remdesivir, Favipiravir, Ribavirin, Tocilizumab, Siltuximab, Fingolimod and Thalidomide are drugs approved to treat other viral infections. The efficiency for treatment of Covid-19 of these drugs is being investigated.

**Figure 1:**
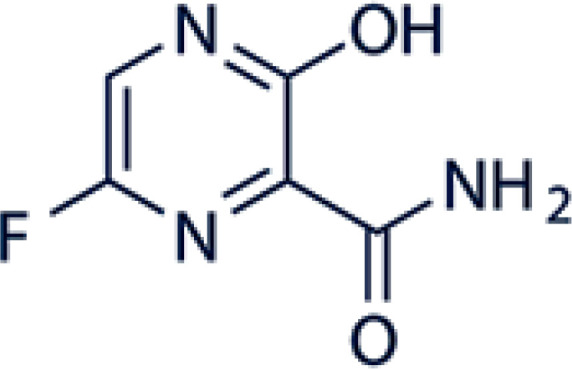
Chemical structure of favipiravir

Favipiravir is an antiviral drug that is active against many RNA viruses, also known as favilavir. Favipiravir (6-fluoro-3-hydroxypyrazine-2-carboxamide) is a pyra-zine analog (Figure 1). The mechanism of action is linked to transcription inhibition and viral gene replication which finally prevents the synthesis of viral *RNA* inside infected cells (3).

It has been shown that favipiravir has an anti-viral effect on several RNA viruses, such as influenza-resistant viruses, bunyaviruses, filoviruses, arenaviruses, Yellow Fever, Rift Valley Fever, Western Equine Encephalitis, West Nile, Mouth and Foot Viruses, Avian Influenza and Norovirus (4-8).

Favipiravir has been assessed in the treatment of Lassa, Ebola, Hanta, and now the Covid-19 virus (9-12) due to its wide range of anti-viral coverage. In an open-label, non-randomized control trial in China, the effectiveness of favipiravir versus LPV/r in Covid-19 was evaluated. Drug safety, viral clearance, and changes in chest CT were investigated in two groups of patients. The results have shown major changes in chest imaging, few adverse effects and a shorter time for viral clearance in the favipiravir group compared with the control group (13).

Several clinical studies are ongoing to assess the benefits of favipiravir in coronavirus disease. The potential candidate drug for Covid-19 disease is considered to be Favipiravir. The key benefits of favipiravir are that it can be delivered orally and given in symptomatic patients but not sufficiently ill to be taken to a hospital. Since many people with mild to moderate Covid-19 patients can be treated at home, this drug can be used in a significant number of patients. However, favipiravir must be administered early after the onset of symptoms to successfully reduce viremia, as with any antiviral drug. Its role in potentially shortening the time of viral spread can also have an epidemiological effect, as viral replication can be reduced at home and in society (14). 

Because of its importance in treating Hanta, Lassa, Ebola, and Covid-19 virus, there is no monograph of favipiravir in USP, EP, and British Pharmacopeia. In addition, there are several methods related to the analysis of favipiravir in pharmaceuticals in the literature. There are two published liquid chromatographic methods for determining favipiravir assay and impurities in active pharmaceutical ingredients (15, 16). A grad-ient liquid chromatographic method was used for chromatographic separation and the run time was 60 min in both of these methods,

Because of its high sensitivity and accuracy, liquid chromatographic is a more widespread method in quality control laboratories. Spectrophotometric method is very simple, as no reagent, pH adjustment or extraction technique is necessary. To this end, a spectrophotometric and an liquid chromatographic method were developed and validated to quantify favipiravir in pharm-aceutical preparations. The findings obtained by these techniques have been statistically compared using variance analysis. They also assessed the reliability and feasibility of these methods focusing on quality control analysis.

## Experimental

*Equipment*

Chromatographic analysis was carried out using an Agilent 1260 series liquid chromatograph equipped with an ultraviolet (UV) detector, a quaternary pump, a vacuum degasser, a column oven, and Chemstation software. The present study also utilized a MettlerToledo electronic balance (Mettler Toledo, Switzerland), a Milli-Q water purification system (Millipore, USA), and UV-Visible spectrophotometer with a double beam using 1.0 cm quartz cells and UVProbe software (Shimadzu UV-1800 spectrophotometer, Japan).

*Chemicals*

In this analysis, analytical grade chemical compounds were used without further purification. Sodium acetate, glacial acetic acid, and acetonitrile were bought from Sigma-Aldrich. Deionized water was purified using a Milli-Q system (Millipore). Pure favipiravir and Favicovir tablets were supplied from Atabay Pharmaceuticals and Fine Chemicals Inc. (Istanbul, Turkey).

*Standard solutions*

To create the calibration curve, the stock standard solution of favipiravir (1000 μg mL^-1^) was prepared in deionized water. The subsequent stock solution has been sonicated and filtered through a 0.22 µm filter. Further, the stock solution was diluted with deionized water to obtain standard solutions at concentrations in the range (10-60 µg mL^-1^) prior to analyses.

*Sample solution*

Ten tablets of favipiravir (Favicovir, 200 mg) have been weighed and crushed into a fine powder. Accurately weighed tablet powder containing 50 mg of favipiravir was transferred to a 50 mL calibrated flask and dissolved in 30 mL of deionized water. The content was shaken for 30 min. The volume was completed with deionized water to get the concentration of 1000 μg mL^-1^. The final solution was filtered using a Whatman filter paper (No. 42)

*Determination of λ*_max_

First, the spectrophotometer was calibrated to zero. Then the maximum absorption wavelength of the favipiravir solution (30 µg mL^-1^) was determined by scanning in the range of 200 and 800 nm.

*Conditions*

Chromatographic analysis was performed on a liquid chromatograph (Agilent 1260) with a UV–vis detector. Favipiravir were analyzed at a flow rate of 1.0 mL min^-1 ^using a mobile phase composed of sodium acetate solution 50 mM (pH 3.0 with glacial acetic acid) and acetonitrile (85:15, v/v). Before use, the mobile phase was filtered and degassed through a 0.22 μm membrane filter. An Inertsil ODS-3 C18 (4.6 mm *×* 250 mm, 5.0 μm particle size) column was used and operated at 30 °C. Favipiravir was detected with the UV detector at 227 nm under room temperature. The run time under these conditions was 10 minutes. UV spectrophotometric method was carried out on a double beam spectrophotometer at 227 nm using 1.0 cm quartz cells for all absorbance measurements.

*Method validation*

Both methods have been validated in compliance with the recommendations of the International Harmonization Conference on the validity of analytical procedures (17, 18). Validation parameters (Specificity, linearity, the limit of detection and quantification, precision, accuracy, and robustness) have been investigated.

*Specificity*

The specificity of both methods was assessed by comparing the spectrums and chromatograms obtained from standard and sample preparations that take part in the pharmaceutical preparations. 

*Linearity*

Standard calibration curves in both methods were obtained by analyzing a series of standard solutions. These standard solutions have been prepared in triplicate, and linearity was assessed using linear regression analysis. 

*Limit of detection and quantification*

Limit of detection and quantification have been determined using the slope of the calibration curve (m) and standard error (s) as displayed in the following equations.

LOD = 3.3 × s/m 

LOQ = 10 × s/m 

*Precision*

The precision of both methods was analyzed in terms of both repeatability (intraday precision) and intermediate precision (interday precision). The repeatability was determined from five replicated injections of a freshly prepared favipiravir solution (30 μg mL^-1^) in the same equipment on the same day. In order to determine intermediate precision, the experiment was also replicated by analyzing the newly prepared solution at the same concentration on three consecutive days. Precision was expressed as R.S.D.% of a series of measurements.

*Accuracy*

The percentage recovery was determined by using three preparations of three different levels of the reference drug of favipiravir. The findings were expressed as the percentage of favipiravir recovered in the sample and R.S.D.%.

*Robustness*

For the liquid chromatographic method, samples were analyzed under different circumstances like changes in mobile phase flow rate (0.9 mL min^-1^ – 1.1 mL min^-1^) and in acetonitrile content (±10%) in the mobile phase and the effect of system suitability parameters have been observed. For the spectrophotometric method, samples have been analyzed under different circumstances like changes in solvents used and detection wavelengths.

*Analysis of marketed formulations*

Freshly prepared stock sample solution diluted with deionized water to obtain sample solution (30 µg mL^-1^). This sample solution was filtered using a filter of 0.22 μm and then analyzed.

## Result and Discussion

*Chromatographic Method*

**Figure 2:**
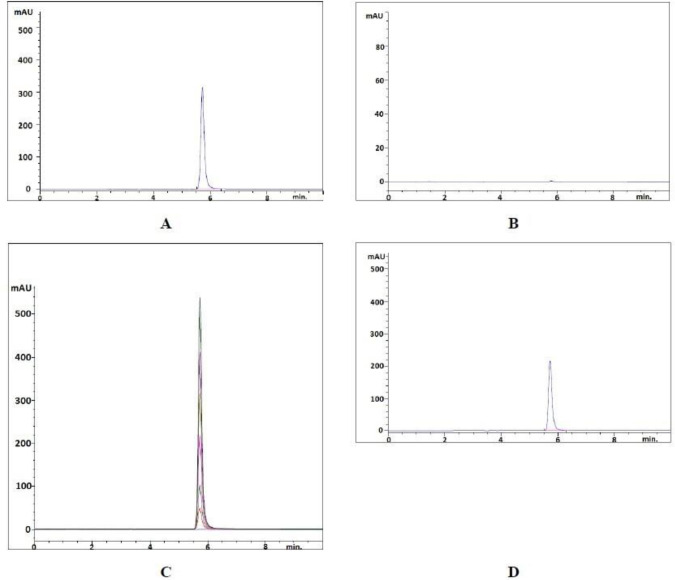
(A) Chromatogram of standard favipiravir (60 μg mL^-1^). (B) Chromatogram of blank solution. (C) Overlay chromatogram (Standard solutions, 10-60 μg mL^-1^). (D) Chromatogram of sample solution (40 μg mL^-1^).

A reversed-phase liquid chromatographic method for estimating favipiravir in pharmaceutical forms has been proposed. In order to get a successful result, chromatographic conditions were adapted. The chromatographic procedure has been optimized to develop an accurate and reproducible method. Different conditions such as mobile phase compositions, different columns and configurations were tested to achieve a sharp peak. The mobile phase was chosen considering the peak parameters (tailing, symmetry), analysis time, easy preparation and cost. Figure 2 displays the chromatogram produced of the favipiravir standard and sample solutions using the developed method. Favipiravir was eluted to form a symmetrical peak, as seen in this figure. The observed retention time (5.725 min) enables the rapid detection of favipiravir, essential for routine research. The resulting favipiravir peak showed that the flow rate of 1.0 mL min^-1^ of the mobile phase consisting of 50 mM acetate buffer (pH adjusted to 3.00) and acetonitrile in the ratio of 85:15 (v/v) on the column used was appropriate. In this developed method, the peak was eluted with a capacity factor of 4.62, a tailing factor of 0.776 and a number of theoretical plates of 11798. 

The equation of the calibration curve was obtained from linear regression analysis of the peak area versus the concentration of favipiravir. Regression equation of the calibration curve for favipiravir was calculated as y = 47.143x + 16.941 at the range of 10-60 μg mL^-1^. The correlation coefficient (r^2^: 0.9999) indicates good linearity and high sensitivity (Table 1). Limit of detection and quantification were 0.40 and 1.10 μg mL^-1^, respectively. 

Repeatability (intraday) and intermediate precision (interday) have determined the precision of this method. Precision was expre-ssed as R.S.D.% of a sequence of measurement. Precision study data were presented in Table 2. The result obtained (R.S.D.%: 0.198) shows a good intra-day precision. Inter-day precision was also calculated from assays on three-day tests, and a mean R.S.D% was 0.204. 

The recovery of the analyte was determined by adding different levels of the standard analyte (80%, 100% and 120%) to the sample solution and analyzing it in the same way. The results of mean percentage recovery, R.S.D.% and standard error were given in Table 3. 

No significant changes in the system suitability parameters were observed when the organic content and flow rate of the mobile phase were changed. Results were presented in Table 4. The low R. S. D.% values showed that the method was sufficiently robust.

*Spectrophotometric method*

The spectrum of a favipiravir solution in water (60 µg mL^-1^) against a blank has been shown in Figures 4A and 4B. Three intense absorbance peaks have been observed at 227, 324, and 363 nm. The most intense absorbance peak (λ_max_) was observed at 227 nm. Several assays were carried out, and the best results have been achieved when using the amplitude from the valley at a wavelength of 227 nm to the zero baseline. The overlay spectrum of favipiravir standard solutions and spectrum of sample solution were given in Figures 4C and 4D. 

Good linearity was achieved in the concentration range of 10-60 µg mL^-1^ of standard solutions of favipiravir (Figure 5). The exact data obtained for the evaluated methods are presented in Table 2. Less than 0.5 of R.S.D.% values have been determined. This shows that both methods provide good sensitivity, but the chromatographic method is more sensitive than the spectrophotometric method. Accuracy was studied using recovery experiments using the methods developed. Both spectrophotometric and chromatographic methods displayed mean recoveries of close to 100 percent, showing adequate accuracy (Table 3).

The method’s robustness was evaluated by testing the effect of minor variations on experimental variables like changes in different solvents and detection wavelengths on the analytical performance. The minor differences in each of the factors did not affect the findings dramatically indicating the developed method for routine analysis is reliable (Table 4). 

*Application of these methods to pharmaceutical preparations*

Both methods developed and validated have been successfully applied for the determination of favipiravir in pharmaceutical formulations. Test results for a tablet containing favipiravir sold in pharmacies were presented in Table 5. The results are very close to the amounts indicated on the label of the tablets. The spectrophotometric and chromatographic methods recommended in this report can be applied appropriately to analyze favipiravir in pharmaceutical preparations.

**Figure 3:**
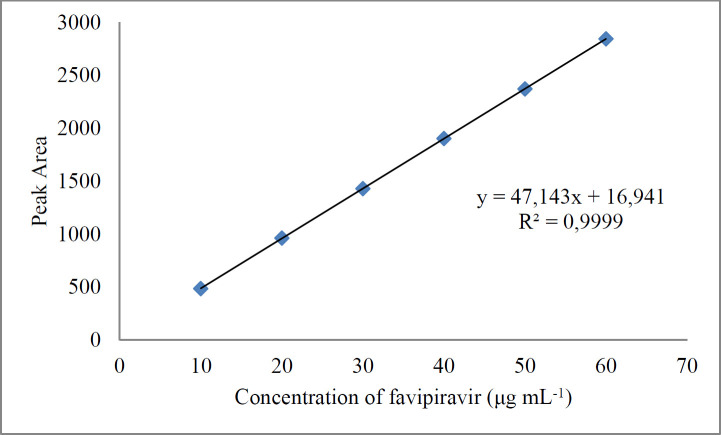
Calibration curve of liquid chromatographic method

**Figure 4:**
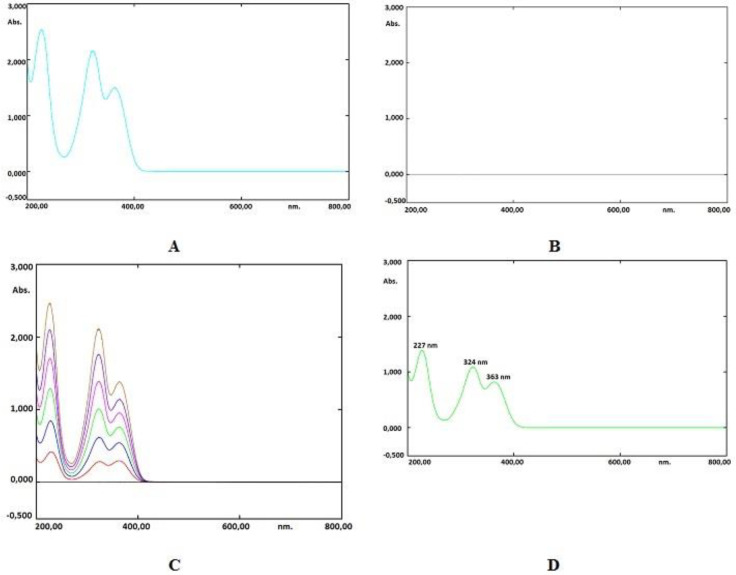
(A) The spectrum of standard favipiravir (60 μg mL^-1^). (B) Spectrum of blank solution. (C) Overlay spectrum (Standard solutions, 10-60 μg mL^-1^). (D) Spectrum of sample solution (30 μg mL^-1^)

**Figure 5:**
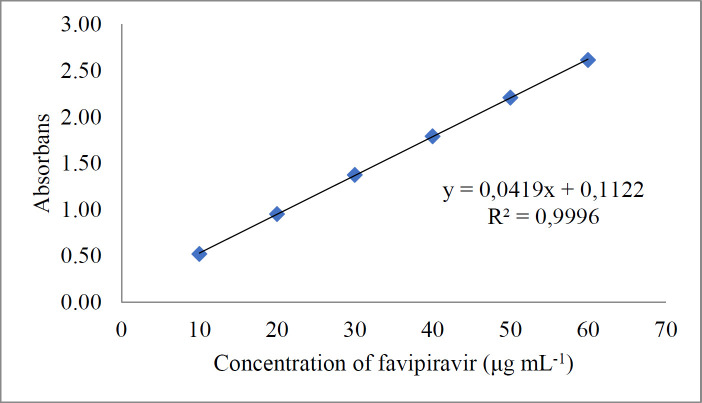
Calibration curve for spectrophotometric method

**Table 1:** Linearity study data

**Parameter**	**Spectrophotometric Method**	**Liquid Chromatographic Method**
Concentration Range (μg mL^-1^)	10-60	10-60
Limit of detection and quantification (μg mL^-1^)	1.4/4.3	0.4/1.1
Slope	0.0419	47.143
Standard Error of Slope	0.00052	0.2700
Intercept	0.1122	16.941
Standard Error of Intercept	0.0073	2.2000
Correlation Coefficient	0.9996	0.9999
Standard deviation of Residuals	1.00	0.29

**Table 2:** Precision tests data

**Precision parameters**	**Spectrophotometric method**		**Liquid chromatographic method**	
	Absorbance*	R.S.D. (%) *	Peak Area*	R.S.D. (%) *
Repeatability	1.375	0.364	1431.17	0.198
Intermediate Precision	1.372	0.372	1430.03	0.204

*(n = 5 for repeatability; n = 15 for Intermediate precision); R.S.D. (%) = Percentage Relative Standard Deviation.

**Table 3:** Recovery tests data

**Methods**	**Level of drug taken**	Mean percent recovery*	R.S.D. (%) *	**S.E.**
**Spectrophotometric method**	80	100.45	0.4781	0.0762
	100	99.83	0.5140	0.2963
	120	100.19	0.5814	0.3350
**Liquid Chromatographic method**	80	100.20	0.4574	0.2646
	100	100.14	0.2908	0.1683
	120	99.87	0.4329	0.3350

*(n = 3); R.S.D. (%) = Percentage Relative Standard Deviation; S.E. = Standard Error.

**Table 4:** Robustness study data

**Method**	**Parameter**	**Value**	**Tailing factor**	**Number of theoretical plates**	**Content (%)**
Liquid Chromatographic method	Acetonitrile composition%	9	0.775	11 824	100.15
		11	0.739	11 789	99.87
	Flow rate (mL min^-1^)	0.9	0.753	11 624	99.78
		1.1	0.746	11 698	100.02
Spectrophotometric method	Solvent	Methanol			100.25
		Ethanol			99.74
		Isopropyl alcohol			100.03
	Detection wavelengths	225			99.94
		229			100.14

**Table 5:** Method application results

		**Spectrophotometric method**		**Liquid Chromatographic method**	
**Formulation **	**Label claim** **(mg)**	**Found favipiravir** **(mg)**	**Assay%** **± S.D.**	**Found favipiravir** **(mg)**	**Assay%** **± S.D.**
Favicovir tablet	200	200.14	100.07 **± **1.00	200.05	100.03 **± **0.29

## Conclusion

Spectrophotometric methods generally do not require complex operations and procedures. It takes less time and is economical. These cases are advantages of the spectrophotometric method over the liquid chromatographic method. Statistically compared, the chromat-ographic method is more precise and accurate than the spectrophotomeric method. The findings suggest that spectrophotometric and chromatographic methods are appropriate methods for quantifying favipiravir in pharmaceutical dosage forms. Excipients in tablets have not interfered with them, and the mobile phase can be prepared very easily. Because both recommended methods are specific, simple, fast, precise and accurate, they can be successfully applied for routine quality control analysis in pharmaceutical dosage forms of favipiravir.
